# Supplementary material for: Clinical practice guideline adherence in oncology: A qualitative study of insights from clinicians in Australia
Source: PLoS One. 2022 Dec 16;17(12):e0279116. doi: 10.1371/journal.pone.0279116 (PMC9757567; doi:10.1371/journal.pone.0279116)
Supplement: S1 Appendix — (DOCX) [file pone.0279116.s002.docx]

**Appendix A: Interview topic guide**

| **Demographic questions for participants to be asked at beginning of survey** | Do you meet the inclusion criteria, have you read the participant information sheet, and have you signed a consent form? If yes, please answer the following questions. If no, please sign a consent form before answering any questions. |
| --- | --- |
|  | What is your age? |
|  | Which LHD do you spend most of your clinical time in? |
|  | Which discipline do you belong to? (Medical oncology, Radiation oncology, Surgery, Other (please specify)  Which cancer stream/s to you predominantly work with? (please specify) |
|  | What is your professional position/seniority currently? (Intern/Resident, Registrar, Advanced trainee, Fellow, Consultant/Staff specialist, Other (please specify)) |
|  | When did you graduate as a specialist (in an oncology field)? |
| **Topic** | **Example questions** |
| Attitudes towards cancer CPGs | 1. *What do you think about cancer treatment CPGs? (e.g., Are they useful? In what way?*  - *Are they useful sources of advice? Are they educational?* - *Are they based on quality interpretation of the evidence? How do you know?* |
| Personal experience using CPGs to guide treatment decision making | 1. *When do you use cancer CPGs to inform your practice? (e.g., for decision support in complex cases, or as standard recommendations in simple cases?)*  - *Is your practice routinely adherent to CPG treatment recommendations? (Why/why not?)* - *What do you think of CPG adherence as a measure of quality of care?* - *Do your colleagues typically adhere to cancer treatment CPG recommendations?* |
| Barriers to adhering to CPG recommendations | 1. *What are the key barriers, if any, that prevent you from adhering to cancer treatment CPG recommendations?*  - *Do CPGs cater for complex cases and take into account patient comorbidities, etc.? (In what way do they/don’t they?)* - *Are patient preferences a facilitator or barrier to CPG adherence?* - *How do you account for patient preference in your use of CPGs in clinical practice? Is the need for individualised care a barrier to CPG adherence?* - *What happens when new evidence is released (e.g., at a conference) that contravenes a CPG?* - *Does the age of the patient or patient comorbidities act as a barrier to adhering to CPG recommendations?* |
| Facilitators that make it easier to adhere to CPG recommendations | 1. *What are the key facilitating factors that enable cancer treatment CPG adherence?*  - *What enables you to adhere to cancer treatment CPG recommendations in your daily practice?* - *When a new CPG is released, what would encourage you to follow the CPG recommendations if they differed to your usual practice?* |
| Improvements needed | 1. *What type of improvements would you like to see in the development of cancer treatment CPGs? (Format? Updates? Feedback?)*  - *How can CPGs be better delivered? What will increase adoption of CPG recommendations, in general? What additional support do you need from CPGs?* |
| Clinical practice variation | 1. *Is variation in clinical practice reasonable, or should there be more conformity? (Why, explain your answer?)*   *Provide examples of cases of CPG adherence rates*  *Is the rate of adherence considered low in those example cases or is it reasonable?* |
