# Supplementary material for: Clinical practice guideline adherence in oncology: A qualitative study of insights from clinicians in Australia
Source: PLoS One. 2022 Dec 16;17(12):e0279116. doi: 10.1371/journal.pone.0279116 (PMC9757567; doi:10.1371/journal.pone.0279116)
Supplement: S2 Appendix — (DOCX) [file pone.0279116.s003.docx]

**Appendix B: Coding Framework**

| Theme 1: CPG content | Applicability of recommendations to patient population |
| --- | --- |
|  | Degree of evidence and level of agreement with evidence underpinning CPGs |
|  | Format- ease of use, references to evidence, and inclusion of patient resources |
|  | How up-to-date CPGs are |
|  | Prescriptiveness of CPG recommendations |
| Theme 2: Individual clinician and patient factors | Clinician personality, and the impact of CPGs on Autonomy |
|  | Generational and disciplinary differences in perceptions towards CPGs |
|  | Litigation concerns |
|  | Patient age, comorbidities, preferences and logistics |
| Theme 3: - Access to, awareness of and availability of CPGs | Access to, awareness of and availability of CPGs |
| Theme 4: Organisational and cultural factors | Access to treatments recommended by CPGs, resource availability and clinician time |
|  | A culture of peer or multidisciplinary review of treatment plans |
|  | Referral pathways |
| Theme 5: Development and implementation factors | Development, adaptations and review of CPGs, by an expert development committee |
|  | CPG Dissemination and Implementation Strategies |
|  | Suggested development and implementation improvements |
