# Supplementary material for: Clinical practice guideline adherence in oncology: A qualitative study of insights from clinicians in Australia
Source: PLoS One. 2022 Dec 16;17(12):e0279116. doi: 10.1371/journal.pone.0279116 (PMC9757567; doi:10.1371/journal.pone.0279116)
Supplement: Attachment — Submitted filename: Response to reviewers_Bierbaum 250722.docx [file pone.0279116.s004.docx]

Dr Anna Ugalde

Editor: PLOS ONE

Manuscript Number PONE-D-22-08374.

25 July 2022

Dear Dr Ugalde

Thank you indeed for the invitation to revise our paper and for the thoughtful comments and feedback. We would like to resubmit the revised manuscript *‘Clinical Practice Guideline adherence in oncology: A qualitative study of insights from clinicians in Australia’* to be considered for publication in PLOS ONE. Please find our response to reviewer comments below.

| Review | Author’s response | Page/line |
| --- | --- | --- |
| **Editor’s comments** |  |  |
| Thanks for the submission to Plos One. This is a very interesting article but as you can see from the reviewer comments there are some concerns about the method and analysis. | Thank you for your consideration of our manuscript and the opportunity for revision. |  |
| I see that the authors have also included a content analysis as well as a thematic; is it worth revising this and focusing on the themes that are present? From the reviewer feedback, potentially the content analysis has confused presentation of the findings. | Thank you for this feedback.  We have removed the content analysis that was presented within the thematic analysis findings (p7-13). The summary of the frequency that codes were discussed, and the trends across clinician specialties, can now be seen at the end of the results section under the subheading Frequency Analysis, p13, and in table 2. | P7-13 |
| Could the authors also ensure that all of the COREQ checklist are addressed? I could see many of them (e.g. gender, training of interviewer) but if this could be assured that would assist. | Please find the COREQ checklist attached. |  |
| Please ensure a clear design statement is clear as this was also identified by the reviewer. | Please find the design statement on page 4. | P4, Methods |
| Could you please also confirm the dates of interviewing are included.  I look forward to your response and reviewing a revised version. | Interviews were conducted between October 2019-January 2020. Please see addition in text, p4 | P4, Recruitment and data collection |
| **Reviewer one’s comments** |  |  |
| Reviewer #1: This is interesting paper, and while the findings are not surprising they remain important to disseminate | We thank the reviewer for their comments. |  |
| The original purpose of EB guidelines was to provide a collation of the best available evidence with recommendations, to "guide" the clinician in making decisions regarding the patient before them. The initial intent was that the clinician would tailor recommendations/guidance to the person sitting before them, taking into account their unique situation. | We agree and believe this is addressed in the manuscript. Please see below where these issues have been discussed in the manuscript:  Discussion - paragraphs 1 and 2, p14.  Results - Theme 1: CPG content  SUBTHEME 1.1: Applicability of recommendations to patient population, p7  SUBTHEME 1.5: Prescriptiveness of CPG recommendations, p9  THEME 2: Individual clinician and patient factors  SUBTHEME 2.4: Patient age, comorbidities, preferences and logistics, p10  To make this even clearer, however, we have modified the introduction – paragraph 1, p3 - to briefly and explicitly include this concept as part of the framing of the research. | P3, Introduction  P14, Discussion  P7, 9, Results  P10 Results |
| As the evidence base has grown to support clinical guidelines, so has evidence for poorer outcomes in some areas when recommendations in guidelines are not followed. There is also variability in the quality of guidelines.  None of these factors have been examined in the paper. The underlying premise appears to be all clinical decisions should be based on EB Guidelines, even though they are a guide". | Again, we agree. These issues are discussed in the following locations in the manuscript:  Results - Theme 1: CPG content  SUBTHEME 1.2: Degree of evidence and level of agreement with evidence underpinning CPGs, p8  SUBTHEME 1.3: Format, ease of use and references to evidence, p8  SUBTHEME 1.4: How up-to-date CPGs are, p8 | P8 |
| A major concern with the paper, while stating it complies with CREQ criteria for reporting qualitative research, is that this is not addressed. | Please see COREQ checklist attached. |  |
| There are significant gaps in information regarding the study design which is not described at all. The findings as presented do not represent thematic analysis, and with out any description of teh research method it is hard to determine what the underpinning philosophical approach or framework was, if there was one. The findings as presented are more in line with content analysis, which would be appropriate in this type of research. As it currently is presented there is concerns about the authors understanding of qualitative research. | Thank you. We have added to the methods section to ensure the methods are clear for the reader. Please see amendments to the Methods section, p4, and p5  See amendments in the results section to the presentation of the thematic analysis findings (p7-13) and summary of the frequency analysis (p13). Just by way of offering reassurance to the reviewer, the authorship team has published a deal of qualitative research over the years. | P4, 5, 7-13 |

Yours sincerely, Mia Bierbaum
